# Efficacy of Electroacupuncture Combined with Methadone Maintenance Therapy: A Case-Control Study

**DOI:** 10.1155/2019/7032581

**Published:** 2019-07-21

**Authors:** Yu-Liang Chen, Tsung-Chieh Lee, Yun-Tai Chen, Lun-Chien Lo, Wen-Yu Hsu, Wen-Chen Ouyang

**Affiliations:** ^1^Department of Traditional Chinese Medicine, Changhua Christian Hospital, Changhua, Taiwan; ^2^College of Chinese Medicine, China Medical University, Taiwan; ^3^Department of Psychiatry, Changhua Christian Hospital, Changhua, Taiwan; ^4^Department of Geriatric Psychiatry, Jianan Psychiatric Center, Ministry of Health and Welfare, Taiwan

## Abstract

High compliance with methadone maintenance therapy (MMT) is crucial to successful opioid abstinence in addicts. However, MMT has numerous side effects, including reductions in quality of life and quality of sleep. Many studies have demonstrated that electroacupuncture relieves withdrawal symptoms in opioid addicts. The present study was a case-control study. From January 2015 to September 2016, 106 patients undergoing MMT at a medical center in central Taiwan were recruited and separated into an electroacupuncture treatment group and a control group. Electroacupuncture was performed for 15 minutes twice weekly for 4 weeks. The electroacupuncture treatment group was discovered to have improved quality of life, especially in terms of vitality and mental health. Although electroacupuncture did not significantly improve sleep quality, we found that sleep quality was significantly improved once methadone dosage had been reduced. Electroacupuncture can improve quality of life in patients undergoing MMT. If methadone dosage can be reduced and electroacupuncture can be employed, both sleep and life quality can be improved.

## 1. Introduction

Drug addiction is a social, economic, and public health problem worldwide. The number of drug addicts in Taiwan has reached approximately 200,000, and drug addiction is most common in young adults [1]. According to the Ministry of Justice, the number of drug-related criminal cases has increased annually since 2004, especially cases of recidivism and combinations of drug offences with other crimes [2]. More than 2,000 drug abuse cases were taken to court in 2014. To increase the withdrawal rate and reduce the relapse rate among drug addicts, adjuvant therapy is crucial. Methadone maintenance therapy (MMT) is a first-line treatment options for people with opiate dependence [3] and was first used in Taiwan in 2006. MMT can reduce the degree of drug dependence [4] and improve social function [5]. High compliance with MMT is critical to successful abstinence from opioids [6]. However, despite its numerous advantages, MMT has many side effects, including constipation, dizziness, fatigue, dry mouth, and gastrointestinal upset; these effects negatively influence compliance [7]. Additionally, 7%-8% of patients undergoing MMT have concurrent physical or psychiatric diseases [8].

Quality of life and sleep quality are of high concern for patients undergoing MMT [9–11]. Routine monitoring of the health status of patients' receiving MMT is recommended [12]. One study revealed that the higher the quality of life of a patient undergoing MMT, the superior his or her MMT treatment outcomes [13]. Quality of life plays a particularly crucial role when treating poly drug users and is related to the recovery process. Quality of life involves social, cultural, and economic contexts and is associated with the outcomes of patients with opioid dependence [14, 15].

Sleep disturbance is related to acute and chronic drug addiction. Opiates disrupt sleep architecture by blocking access to rapid eye movement sleep and to the deeper restorative stages of nonrapid eye movement sleep [16]. Sleep quality is one aspect of and closely related to quality of life [17]. One study discovered that more than half of a group of patients receiving MMT reported sleep disturbance [18]. Sleep disturbance caused by MMT is one reason why patients do not complete a course of MMT [7]. The 1-month prevalence of sleep disturbance in patients with heroin dependence was high in one study and associated with greater dependence, depression, and poorer physical-health-related quality of life [19].

Traditional Chinese Medicine (TCM) is the most commonly used therapy in Asia. TCM has a history dating back thousands of years, and its most well-known treatment is acupuncture. The World Health Organization identifies many indications for acupuncture, including drug addiction [20]. Some studies have discussed the efficacy of acupuncture for helping people quit smoking or give up alcohol [21]. The effect of acupuncture for treating drug addiction has been confirmed [22]. Acupuncture can relieve the withdrawal symptoms of heroin addicts [23]. One meta-analysis demonstrated that acupuncture combined with MMT can lessen withdrawal symptoms; however, the effect was not sustained over 6 months [24]. Acupuncture is simple, convenient, and inexpensive and has few side effects [25]. Many studies have reported positive effects of electroacupuncture in treating opiate withdrawal symptoms [26, 27]. Thus, the present study employed electroacupuncture to treat patients undergoing MMT and determine whether improvements were consequently achieved in quality of life, sleep quality, and the drug abstinence success rate.

## 2. Patients and Methods

### 2.1. Patients

We recruited patients who underwent MMT at a psychiatric clinic in one medical center in central Taiwan between June 2016 and May 2017. The patients were diagnosed as having opioid addiction and received MMT for more than 30 days. This was a case-control study, and the patients could choose whether to undergo electroacupuncture. They continued to receive MMT during the research period. A total of 106 patients undergoing MMT were invited to participate in this study. One patient dropped out. The study was approved by Changhua Christian Hospital Institutional Review Board (IRB No. 160320).

### 2.2. Methods and Data Collection

The patients who chose to undergo electroacupuncture treatment composed the treatment group, and those who chose otherwise formed the control group. The treatment group received 15 minutes of electroacupuncture treatment twice weekly for 4 weeks. The electroacupuncture were performed at the bilateral Hegu (LI-4) and Zusanli (ST-36) acupoints. “DeQi” was requested (manifesting as soreness, numbness, heaviness, and distension; sensations that are believed to reflect the efficacy of the treatment) first and then applied electrical stimulation. The needles were left for 15 minutes and then removed. Electrical stimulation can be used for the ear and body points at a frequency of 100 Hz at low intensity for 10–15 minutes in order to achieve an enhanced response [28]. The needles were using stainless steel “Tennyson” disposable acupuncture needle (N3215, Tennyson medical instrument developing company limited, Taiwan). We applied portable “Ming Horng” low-frequency stimulator (Model MH-350, 5 channel TENS unit and electrical needle stimulator, Ming Horng industrial Company limited, Taiwan) as the electric stimulation machine. The frequency of stimulation alternated between 20 and 100 Hz (dense and disperse, DD) at automatic 2-second intervals, and the maximum intensity was 1 mA. Dosage of methadone was recorded before and after treatment finished. The Short-Form Health Survey (SF-36) was used to assess quality of life, and the Pittsburgh Sleep Quality Index (PSQI) was used to evaluate sleep quality. The SF-36 is a widely used health status measure and is reportedly reliable and valid for surveying the health status of patients with chronic medical conditions such as hypertension, myocardial infarction, congestive heart failure, arthritis, and depression [29]. The SF-36 has eight aspects: physical functioning, role limitations of physical problems, bodily pain (BP), general health (GH), vitality (“VT” hereafter), social functioning, role of emotions (RE), and mental health (MH). The scores for each aspect are calculated and transformed into a score ranging from 0 to 100. The higher the score, the better the health status of the individual in question [30]. The Taiwanese version of the PSQI [31] is reportedly reliable and valid for evaluating sleep quality [32]. The 19 PSQI items are categorized into seven aspects: subjective sleep quality (SSQ), sleep latency (SL), sleep duration (SD), habitual sleep efficiency, sleep disturbance (“S Db” hereafter), use of sleep-related medication (USM), and daytime dysfunction (DD). Each aspect is rated on a 4-point scale (from 0 to 3, with 3 indicating a more profound effect), and the aspect scores are summed to yield a global score (from 0 to 21). A higher score indicates greater severity of S Db, and a global score > 5 indicates poor sleep [31].

### 2.3. Statistical Analysis

Data analysis was performed using the SPSS/PC statistical software package, version 22.0 (Chicago, IL, USA). The methods employed were descriptive statistics, t tests, Pearson product-moment correlation coefficient calculation, and logistic regression. P < 0.05 indicated statistical significance.

## 3. Results

### 3.1. Patient Characteristics

Table 1 details the group distribution and characteristics of the enrolled patients. A total of 107 patients undergoing MMT were invited into this study. One patient dropped out during treatment. The treatment group comprised the 76 patients who accepted electroacupuncture, whereas the control group comprised the 30 patients who did not. The average age of the treatment group was higher than that of the control group (48.88 vs. 45.80 years). The sex ratios of the two groups were similar, with each group having more male than female patients. Regarding educational level, the most common group was those with a junior high school level of education (50% vs. 47%); 32% of the treatment group had a primary school educational level, compared with 10% in the control group. Approximately 40% of the patients were married; most were unmarried (single, divorced, or widowed).

### 3.2. Improvement in Quality of Life

**Figure 1:**
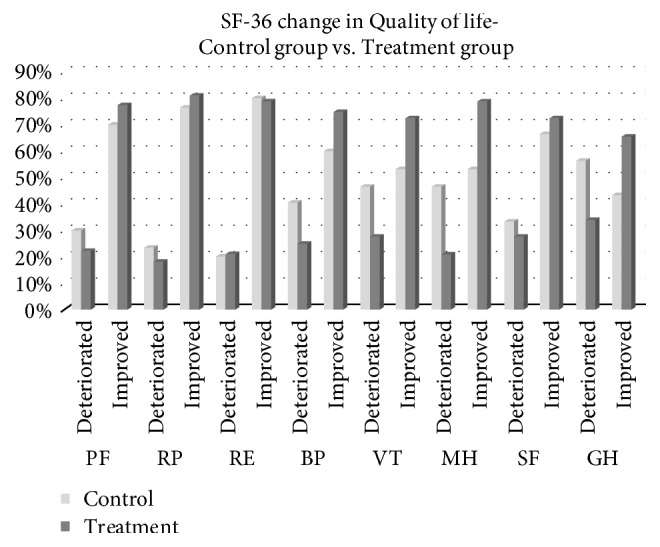
Improvement in quality of life. PF: physical functioning; RP: role limitations of physical problems; BP: bodily pain; GH: general health; VT: vitality; SF: social functioning; RE: role of emotions; MH: mental health.

Figure 1 plots the improvement and deterioration in SF-36 scores for the two groups. The treatment group exhibited improvements in most aspects, especially VT, MH, GH, and BP. By contrast, the control group exhibited deterioration in most aspects. Thus, the results revealed that the posttest scores were improved in the treatment group but not the control group.

### 3.3. Influence of Electroacupuncture on Quality of Life

To evaluate the efficacy of electroacupuncture, we used improvements in SF-36 scores as an index to assess the influences of electroacupuncture and methadone dosage. We included multiple factors—age, sex, educational level, and marital status—for adjustment. Logistic regression was performed, and the results are presented in Table 2. Except for the RE aspect, which deteriorated, all aspects improved. The odds ratios of VT and MH in the treatment group versus the control group indicated significant improvements in SF-36 scores.

### 3.4. Improvement in Sleep Quality

**Figure 2:**
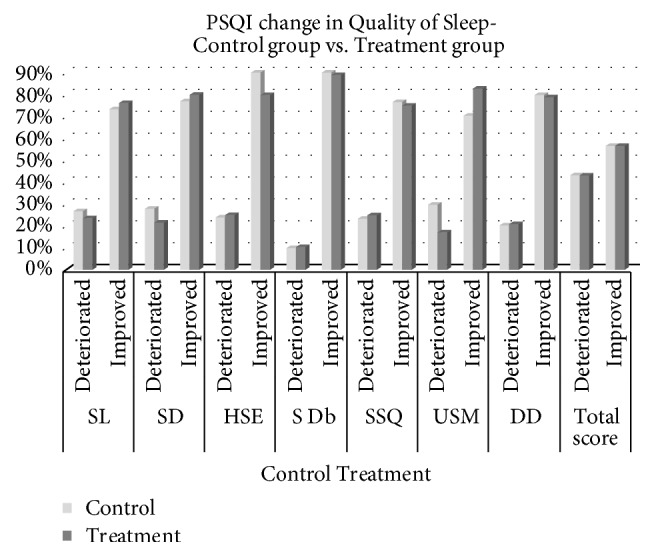
Improvement in sleep quality. SSQ: subjective sleep quality; SL: sleep latency; SD: sleep duration; HSE: habitual sleep efficiency; S Db: sleep disturbance; USM: use of sleep-related medication; DD: daytime dysfunction.

Figure 2 illustrates improvements in sleep quality in both groups. The results revealed little improvement in SL, SD, and USM. In general, no significant differences were discovered between the two groups.

### 3.5. Influence of Electroacupuncture on Sleep Quality

To evaluate the efficacy of electroacupuncture, we used improvements in PSQI scores as an index to assess the influences of electroacupuncture and methadone dosage. We again included many factors—age, sex, educational level, and marital status—for adjustment. Logistic regression was performed, and the results are displayed in Table 3. No significantly different improvements were observed between the treatment and control groups. However, heroin dosage decrease had significance in terms of improvement of the total PSQI score compared to heroin dosage remain. This result showed that heroin dosage decrease has better sleep quality than heroin dosage remain.

## 4. Discussion

The objective of this study was to evaluate the effects of electroacupuncture in patients undergoing MMT. By comparing the pretest and posttest scores of the treatment and control groups, we intended to identify the main factors affecting improvement in quality of life and sleep quality. This study revealed that electroacupuncture can improve quality of life, especially VT and MH. Contrary to our prior expectations, we also discovered that decrease in methadone dosage was the only factor that improved sleep quality.

Although TCM is widely used in Taiwan, scientific research is required to obtain more evidence regarding its effectiveness. In accordance with previously obtained evidence, we enrolled patients with drug addiction who were undergoing Western medicine treatment as the study subjects. Evidence has shown that methadone is beneficial when drug addicts attempt to abstain; however, the advantages of TCM have not yet been proven. We attempted to elucidate the effect of combining Western and Chinese medicine on drug abstinence in this study. We evaluated the effect of Western medicine alone and in combination with TCM. According to the results of our study, quality of life can be improved when MMT is combined with TCM. The results regarding sleep quality before and after drug abstinence can serve as a reference for future research.

Regarding the aspects of quality of life, electroacupuncture significantly improved VT and MH in the enrolled patients undergoing MMT. One study demonstrated that substance dependence is a defense mechanism; through substance use, a user can escape persistent negative emotions [33]. Substance abusers depend on their drugs of choice to relieve or treat particular negative emotions [33]. Narcotics and hypnotics are deployed against rage, shame, jealousy, and particularly anxiety related to these feelings; stimulants are used against depression and weakness; psychedelics are used against boredom and disillusionment; and alcohol is deployed against guilt, loneliness, and related anxiety. A review of the literature indicated that MH is closely related to substance dependence. If TCM intervention can prevent negative emotions caused by drug abuse, an increased drug abstinence rate is expected. Another mechanism is action through endogenous opiates and their receptors. Low-frequency electroacupuncture (2 Hz) accelerates the release of *β*-endorphin, endomorphin, and encephalin, all of which interact with *μ* and *δ* opioid receptors. By contrast, high-frequency electroacupuncture (100 Hz) accelerates the release of dynorphin, which interacts with *κ* opioid receptors [34–37]. The effects of electroacupuncture are relief from painful conditions, aid in drug withdrawal, and production of other physiological effects [37].

One systematic review [38] provided evidence of the positive effects of acupuncture in the treatment of opiate addiction; this review included studies on electroacupuncture, manual acupuncture, Han's acupoint nerve stimulation, and auricular acupuncture. The most frequently used acupoints, Zusanli (ST36) and Hegu (LI4), reportedly have positive effects in the treatment of opiate addiction. The present study provided an objective assessment of the effect of electroacupuncture combined with Western medicine; the results could increase confidence regarding TCM's benefits.

Regarding sleep quality, we discovered no significant improvement after electroacupuncture treatment. A decrease of heroin dosage did, however, improve general sleep quality. Opiates disrupt sleep architecture by blocking access to rapid eye movement sleep and the deeper restorative stages of nonrapid eye movement sleep [17]. Opiates decrease adenosine levels in two critical areas that modulate arousal state: the pontine reticular formation and the substantia innominate within the basal forebrain. Decrease of heroin dosage can reduce sleep architecture disruption. One study employed electroacupuncture plus auricular acupuncture to treat patients undergoing MMT [27]. The posttreatment PSQI scores of the true acupuncture group were significantly improved in terms of SSQ, SL, and DD and in the total scores, but no such improvements were discovered in the sham acupuncture group; the patients still experienced insomnia despite true acupuncture treatment. Activation of the endogenous opioid system through acupuncture may help to reduce heroin cravings and thus improve sleep quality. The present study was valuable because it employed logistic regression and odds ratio analyses. The results demonstrated that electroacupuncture did not significantly improve sleep quality. Decrease in heroin dosage was the primary factor affecting sleep quality. Opiates directly influence sleep quality; thus, administering electroacupuncture in patients undergoing MMT was the advantage of this study.

This study had some limitations. First, the 4-week electroacupuncture course was insufficient; the TCM treatment did not have enough time to exert its full effect. Second, the study patients were recruited through our MMT outpatient department and were restricted to those willing to participate. They may have had high expectations regarding the effectiveness of electroacupuncture, leading to the placebo effect. To further evaluate the effect of electroacupuncture, larger controlled studies employing long-term treatment protocols plus sham acupuncture are required.

Despite the aforementioned limitations, this study provided a safe, convenient treatment method with few side effects through selection of specific acupoints. Twice weekly electroacupuncture treatment for 4 weeks was widely accepted by most of the participants.

## 5. Conclusion

Electroacupuncture is beneficial because it improves opioid abstinence. The results of this study suggest that electroacupuncture can improve quality of life; however, no evidence regarding the positive effect of electroacupuncture on sleep quality was obtained. Electroacupuncture is a safe, widely accepted, and potential nonpharmacological intervention for improving opioid abstinence.

## Figures and Tables

**Table 1:** Baseline characteristics.

		Control group (n=30)		Treatment group (n=76)		
		n	%	n	%	P value
Age	mean±Std	45.80±9.25		48.88±8.98		.117
Gender	Male	26	87%	65	86%	.879
	Female	4	13%	11	14%	
Education	Elementary School	3	10%	24	32%	.010
	Junior High School	14	47%	38	50%	
	Senior High School	13	43%	14	18%	
Marital Status	Unmarried	11	37%	25	33%	.642
	Married	13	43%	29	38%	
	Divorced or Widowed	6	20%	22	29%	

**Table 2:** Influence of electroacupuncture on SF-36 scores.

Aspects (Deteriorated vs. Improved)	Independent Variable	Odds ratio	95% CI		P value
Physical Functioning	Treatment vs. Control	1.23	0.42	3.59	.708
	Heroin dosage increase vs. remain	1.58	0.45	5.58	.478
	Heroin dosage decrease vs. remain	2.35	0.73	7.58	.152
Role Physical	Treatment vs. Control	1.33	0.44	4.06	.612
	Heroin dosage increase vs. remain	1.17	0.31	4.37	.816
	Heroin dosage decrease vs. remain	1.83	0.54	6.23	.332
Role Emotional	Treatment vs. Control	0.92	0.30	2.89	.893
	Heroin dosage increase vs. remain	0.80	0.18	3.58	.767
	Heroin dosage decrease vs. remain	0.69	0.19	2.58	.582
Bodily Pain	Treatment vs. Control	2.10	0.78	5.67	.143
	Heroin dosage increase vs. remain	2.98	0.87	10.24	.083
	Heroin dosage decrease vs. remain	2.37	0.79	7.11	.123
Vitality	Treatment vs. Control	2.70	1.03	7.05	.043
	Heroin dosage increase vs. remain	1.40	0.42	4.64	.587
	Heroin dosage decrease vs. remain	1.00	0.34	2.97	.994
Mental Health	Treatment vs. Control	3.72	1.37	10.12	.010
	Heroin dosage increase vs. remain	0.93	0.25	3.53	.916
	Heroin dosage decrease vs. remain	0.60	0.18	1.99	.408
Social Functioning	Treatment vs. Control	1.38	0.52	3.68	.517
	Heroin dosage increase vs. remain	1.15	0.34	3.97	.821
	Heroin dosage decrease vs. remain	0.98	0.33	2.94	.970
General Health Perception	Treatment vs. Control	2.40	0.95	6.09	.064
	Heroin dosage increase vs. remain	0.90	0.28	2.91	.862
	Heroin dosage decrease vs. remain	1.00	0.35	2.87	.998

Control variables: age, sex, educational level, and marital status.

**Table 3:** Influence of electroacupuncture on PSQI scores.

Aspects (Deteriorated vs. Improved)	Independent Variable	Odds ratio	95% CI		P value
Sleep latency	Treatment vs. Control	1.26	0.42	3.59	.672
	Heroin dosage increase vs. remain	0.57	0.15	2.17	.409
	Heroin dosage decrease vs. remain	0.75	0.22	2.54	.638
Sleep duration	Treatment vs. Control	1.18	0.40	3.45	.763
	Heroin dosage increase vs. remain	0.51	0.11	2.34	.389
	Heroin dosage decrease vs. remain	0.51	0.12	2.10	.350
Habitual sleep efficiency	Treatment vs. Control	0.46	0.11	1.96	.292
	Heroin dosage increase vs. remain	0.35	0.07	1.71	.196
	Heroin dosage decrease vs. remain	1.47	0.30	7.26	.636
Sleep disturbance	Treatment vs. Control	0.55	0.10	3.01	.487
	Heroin dosage increase vs. remain	1.05	0.09	12.98	.968
	Heroin dosage decrease vs. remain	0.35	0.05	2.73	.318
Subjective sleep quality	Treatment vs. Control	0.97	0.33	2.86	.954
	Heroin dosage increase vs. remain	1.45	0.38	5.46	.583
	Heroin dosage decrease vs. remain	1.11	0.36	3.45	.851
Use of sleep medication	Treatment vs. Control	1.34	0.42	4.27	.623
	Heroin dosage increase vs. remain	2.21	0.52	9.38	.281
	Heroin dosage decrease vs. remain	1.69	0.49	5.85	.405
Daytime Dysfunction	Treatment vs. Control	0.79	0.22	2.84	.720
	Heroin dosage increase vs. remain	0.37	0.07	1.81	.218
	Heroin dosage decrease vs. remain	0.79	0.18	3.37	.748
Total score	Treatment vs. Control	1.11	0.43	2.87	.836
	Heroin dosage increase vs. remain	1.42	0.45	4.51	.551
	Heroin dosage decrease vs. remain	3.40	1.16	9.97	.026

Control variables: age, sex, educational level, and marital status.

## Data Availability

The data belongs to Department of Traditional Chinese Medicine, Changhua Christian Hospital, Changhua, Taiwan.
